# Association Between Depression and the Short Sleep Duration on Weekdays and Weekends in South Korean Adults: A Cross-Sectional Study Using the 2016 and 2018 Korea National Health and Nutrition Examination Survey

**DOI:** 10.3390/healthcare13091043

**Published:** 2025-05-01

**Authors:** Mi-Jung Eum, Euna Si

**Affiliations:** Department of Nursing, Gangdong University, Eumseong-gun 27710, Chungcheongbuk-do, Republic of Korea; emj44@hanmail.net

**Keywords:** sleep, sleep duration, depression, cross-sectional studies, patient health questionnaire-9 (PHQ-9)

## Abstract

Objective: This study aimed to examine the association between sleep duration on weekdays and weekends and depression among Korean adults. Design: Cross-sectional study. Setting: South Korea, utilizing data from 2016 and 2018 Korea National Health and Nutrition Examination Survey (KNHANES). Participants: A total of 7669 South Korean adults aged 19−64 years. Measures: Depression was assessed using the Patient Health Questionnaire-9 (PHQ-9). Sleep duration was classified separately for weekdays and weekends and categorized into three groups: <7, 7–9, and ≥9 h. Multiple logistic regression analysis was performed, adjusting for socioeconomic, lifestyle, and health-related variables. Results: Among the participants, 3.6% were identified as having depression. On weekdays, 40.5% of respondents reported an average sleep duration of less than 7 h per day, whereas only 20.3% did so on weekends. Compared to those who averaged 7 to less than 9 h of sleep per day, individuals with short sleep duration (<7 h) on weekdays had significantly higher odds of depression (OR: 1.65; 95% CI: 1.16–2.35), as did those with short sleep on weekends (OR: 1.62; 95% CI: 1.10–2.38). Notably, participants who consistently averaged less than 7 h of sleep per day across both weekdays and weekends demonstrated the highest odds of depression (OR: 1.76; 95% CI: 1.13–2.75). Conclusions: Short sleep duration on both weekdays and weekends was found to be associated with depression. These findings suggest that sleep duration should be considered an important factor in the prevention of depression.

## 1. Introduction

Depression is a common psychiatric disorder affecting over 260 million people worldwide, with the global burden of mental illness steadily increasing [1]. In the United States, its prevalence rose significantly between 2015 and 2020, particularly among younger individuals [2]. Similarly, data from South Korea’s Health Insurance Review and Assessment Service (HIRA) in 2022 reported a continuous five-year increase in mental health treatment, primarily for major depressive disorder (MDD) [3]. Persistent or worsening depression is associated with functional impairment, increased morbidity, and a higher risk of mortality [2,4], and, due to its association with suicide, it is considered a significant public health issue distinct from normal mood fluctuations [1].

Depression is influenced by a combination of factors, including genetic predisposition, psychological stress, interpersonal difficulties, and physical illnesses [5,6,7]. Notably, disruptions in biological rhythms and poor sleep quality play a significant role in both the onset and progression of depression [8]. Recently, the increasing prevalence of disturbances in the sleep–wake cycle has made this issue more critical.

According to the National Sleep Foundation, adults are recommended to get 7–9 h of sleep per day [9]. However, in recent decades, global sleep duration has decreased [10], with a significant reduction in sleep duration among U.S. adults since 2013 [11,12]. According to the Organization for Economic Co-operation and Development (OECD), the average sleep duration of Koreans is the shortest, recorded at approximately 6.8 h in 2019 [13,14]. Additionally, data from the National Health Insurance Service (NHIS) indicate a rising trend in the number of individuals diagnosed as having sleep disorders, with the prevalence in women being 1.4 times higher than in men [15]. Specifically, the 2019 World Sleep Survey revealed that Koreans often experience sleep deprivation during weekdays (average: 6.7 h) and compensate for this by getting more sleep on weekends (average: 7.8 h) [16].

Sleep plays a vital role in health by contributing to metabolic regulation, immune and endocrine functions, and physical performance [17,18]. Insufficient sleep duration or poor sleep quality disrupts circadian rhythms, increases fatigue, and elevates the risk of obesity, hypertension, diabetes, cognitive impairment, and cardiovascular diseases [19,20,21,22,23]. Ultimately, these adverse outcomes may contribute to an increased risk of mortality [24].

Individuals who maintained an appropriate sleep duration (7–9 h) demonstrated better health-related quality of life and a 19% lower risk of premature mortality [25]. In contrast, insufficient or excessive sleep has been associated with increased stress, depressive symptoms, and a potential need for psychiatric intervention [26]. Abnormal sleep patterns and sleep disorders are closely linked to depression [27], and not only short sleep durations on both weekdays and weekends [28], but also large discrepancies in sleep duration between weekdays and weekends have been shown to negatively affect health [29]. These findings suggest the importance of examining not only average sleep duration but also weekday–weekend sleep patterns.

Although the relationship between sleep and both physical and mental health has been widely studied, the findings remain inconsistent. These inconsistencies may arise from differences in study populations (e.g., sex, age, race, occupation), research designs, or definitions of sleep duration. Moreover, psychological distress may be influenced by other factors such as hormonal changes, comorbid conditions, and limited social support [30]. Given the critical role of adequate sleep in maintaining overall well-being, its impact on mental health deserves attention. Nevertheless, studies investigating the association between average sleep duration across weekdays and weekends and related health outcomes remain scarce.

In this study, we hypothesize that there is a significant association between sleep duration and depression and aim to examine this relationship using data from the 7th Korea National Health and Nutrition Examination Survey (KNHANES) conducted in 2016 and 2018, targeting adults aged 19 to 64 years.

## 2. Materials and Methods 

### 2.1. Study Design

This cross-sectional study utilized raw data from the 7th KNHANES conducted in 2016 and 2018 [31]. Information on average sleep duration on weekdays and weekends was collected only intermittently, specifically in 2016–2018 and 2021. However, 2021 data were excluded from this study to minimize potential confounding effects and ensure the validity of the findings, as they were considered to have been influenced by the COVID-19 pandemic. According to the Philips 2021 Global Sleep Survey, 70% of respondents reported experiencing new sleep challenges, and 37% indicated that the COVID-19 pandemic adversely affected their sleep quality [32]. Most studies on mental health during the COVID-19 pandemic have reported its significant negative impact, including increased depression, anxiety, and psychological distress [33,34,35].

### 2.2. Study Population

Data from the most recent Population and Housing Census that were available during the sampling were utilized as a sampling framework for the KNHANES to make a representative sample of people aged ≥19 years living in Korea for the target population. The study population was constructed using the most recent available data from the Population and Housing Census as a sampling framework to create a representative sample of individuals aged ≥19 years living in Korea.

For the primary and secondary sampling of constituencies and households, two-stage stratified cluster sampling was used. The health survey and physical examination survey were conducted in mobile screening centers, and each household was personally visited to conduct the nutrition survey. Participants were interviewed to acquire information on education, economic activity, morbidity, and healthcare use in the health survey and every item in the nutrition survey; for health behaviors, such as smoking and alcohol use, in the health survey, a self-administered questionnaire was used. The physical examination survey involved personal measurement, observation, and specimen analysis.

Our study population initially consisted of 16,142 individuals. However, we excluded those aged <19 and >65 years (*n* = 6556), individuals diagnosed with cancer, cardiovascular disease, or thyroid disease, which can affect sleep and depression (*n* = 1040) [36,37], those diagnosed with depression (*n* = 519), and participants with missing data in the questionnaire (*n* = 358). Ultimately, 7669 individuals were included in the analysis.

### 2.3. Measurements and Variable Definitions 

#### 2.3.1. Depression

Although the 7th KNHANES contains raw data from 2016 to 2018, the Patient Health Questionnaire-9 (PHQ-9) survey was only conducted in 2016 and 2018; thus, our study only utilized the data obtained from 2016 and 2018.

PHQ-9 is highly sensitive and specific, thereby practical for diagnosing major depression disorders [38]. PHQ-9 is a self-reporting questionnaire consisting of nine question items, namely, “little interest or pleasure in working”, “depressive mood”, “sleep disorder”, “fatigue and lack of energy”, “poor appetite or overeating”, “unreasonable or inadequate guilt”, “difficult to concentrate on things”, “dullness and slowness or impatience”, and “thoughts on suicide”. The measurement consists of a four-point Likert scale from 0 to 3, and the total score is from 0 to 27; the higher the score, the higher the depression. The survey results led to the following classifications: 0–4, no depression; 5–9, mild depression; 10–14, moderate depression; 15–19, moderately severe depression; and ≥20, severe depression. Based on the literature review [38] and the Korea Centers for Disease Control and Prevention (KCDC)’s formula for calculating the prevalence of depression, the PHQ-9 score of 10 points was set as the cutoff value [31]. Therefore, a PHQ-9 score of 10–27 points indicated depression.

#### 2.3.2. Sleep Duration

A self-administered questionnaire was used for examining the sleep duration, which was divided into “average sleep duration on weekdays” and “average sleep duration on weekends”. The average sleep duration on weekdays was classified into <7, 7–<9, and ≥9 h, according to the answers to the question “What time do you usually go to bed and get up on weekdays (or work days)?” Likewise, the average sleep duration on weekends was categorized into <7, 7–<9, and ≥9 h, according to the answers to the question “ What time do you usually go to bed and get up on weekends (or non-work days, days before non-work days)?” The classification of sleep duration conformed to the standards of the American Sleep Foundation [9] and the literature review [39,40].

#### 2.3.3. Other Independent Variables 

The socioeconomic factors included gender (male, female), age (19–29, 30–39, 40–49, 50–59, and ≥60 years), education (elementary school graduate or lower education levels, middle school graduate, high school graduate, and college graduate or higher education levels), income level (1st, 2nd, 3rd, and 4th quartiles), marital status (married, single), and economic activity (yes, no).

The lifestyle factors included smoking (current smoker, ex-smoker, and nonsmoker), alcohol use (high-risk alcohol, proper alcohol, and no alcohol use). The high-risk alcohol use group included a round of ≥7 glasses (or about 5 cans of beer) for men and ≥5 glasses (or about 5 cans of beer) for women and ≥2 rounds of drinking per week. Furthermore, the participants were asked to answer “yes” or “no” to the question about physical activity according to the intensity and frequency of weekly exercise.

The body mass index (BMI), activity limitation (yes, no), stress (much, little), subjective health status (good, average, bad), and chronic disease status (yes, no) constituted the health-related factors.

For the BMI, <18.5, 18.5–25, and ≥25 kg/m^2^ indicated underweight, normal weight, and obesity, respectively. Participants who answered “yes” to activity limitations reported feeling restricted in activities of daily living and social activities due to health problems or physical/mental disorders. Participants’ chronic diseases are those with more than one of the hypertension, diabetes, and dyslipidemia diagnosed by doctors.

### 2.4. Statistical Analysis

When using raw data from the Korea National Health and Nutrition Examination Survey (KNHANES), appropriate sampling weights must be applied to account for the complex survey design. These weights are determined based on strata, clusters, and various survey components, domains, and items. Researchers are advised to choose the appropriate type of weight according to the objective of their analysis and apply it prior to performing statistical procedures. The KNHANES employs a two-stage stratified cluster sampling design rather than simple random sampling. Therefore, it is strongly recommended that statistical analyses reflect this complex survey structure. In accordance with the guidelines provided by the KCDC, all analyses in this study were conducted using a complex sample design with sampling weights applied prior to analysis.

Categorical variables were expressed in frequency and percentage. The prevalence of depression based on the participants’ characteristics and the relationship such characteristics were examined using the chi-square test. The average sleep duration on weekdays and weekends was divided into <7, 7–<9, and ≥9 h. The significant variables in the chi-square test were controlled as corrected variables for the multiple logistic regression analysis.

To investigate the association between depression and average sleep duration on weekdays and weekends, we computed the multiple logistic regression model to estimate the adjusted odds ratios (aORs) and their corresponding 95% confidence intervals (CIs), with the PHQ-9 score of <10 as the reference category. In Model I, adjustments were made for demographic variables including age and sex. Model II further accounted for socioeconomic and behavioral factors, namely educational level, income, marital status, employment status, smoking, and alcohol use. Model III extended the adjustment by incorporating health-related variables such as body mass index (BMI), activity limitation, perceived stress, self-rated health, and chronic disease status. All statistical data were analyzed using the SPSS program, version 23 (IBM Corp., Armonk, NY, USA), and *p* < 0.05 indicated a statistically significant difference in the two-sided test.

### 2.5. Ethical Consideration

All versions of the KNHANES were executed by the KCDC. Every subject was asked to give an informed consent and was kept anonymous before every analysis was completed. The protocol of the present study was conducted with the approval of the Institutional Review Board of the Catholic University of Korea (MIRB-MYUN20200221-001).

## 3. Results

### 3.1. Depression by Participants’ Characteristics

Table 1 shows the relationship between depression and the participants’ general characteristics. The mean age was 41.2 ± 0.22 years, and 272 out of 7669 participants (3.6%) had depression. The average sleep duration on weekdays was 6.96 ± 0.17 h, whereas that on weekends was 7.78 ± 0.20 h. On an average, participants slept 49.2 min more on weekends than on weekdays.

Depression was most likely observed among those who were 19−29 age, elementary school graduates and who were in the 1st quartile of income. The unmarried were more depressed than the married. Current smokers, high-risk alcohol users, and those without economic activity were also more likely to have depression, as well as those with activity limitation and chronic diseases.

Those with high stress and poor subjective health status had a significantly high chance of depression. In addition, 4.5% of the participants who slept <7 h a day on average on weekdays and 6.3% of those on weekends had depression. According to the chi-square test results, all of the variables, except physical activity, were significantly different.

### 3.2. Participants’ General Characteristics According to Sleep Duration on Weekdays and Weekends

Table 2 summarizes participants’ general characteristics according to sleep duration on weekdays and weekends. Out of the 7669 participants, 40.5% slept <7 h a day on average on weekdays, and most of them were women, elementary school graduates or at a lower education level, and in the 4th quartile of income. In addition, those who had activity limitation, chronic diseases, high stress, and poor subjective health status were more likely to have insufficient sleep.

On weekends, 20.3% participants slept <7 h a day on average. Most of them were elementary school graduates or at a lower education level. Likewise, those who had activity limitation, chronic diseases, high stress, and poor subjective health status were more likely to have inadequate sleep. 

### 3.3. Multiple Logistic Regression Analysis of the Association Between Depression and the Sleep Duration on Weekdays and Weekends

Table 3 presents the results of a multiple logistic regression analysis examining the association between depression and sleep duration on weekdays and weekends. To explore this relationship, we conducted a stepwise regression analysis, progressively adjusting for potential confounding variables across three models.

In Model I, adjustments were made for demographic variables including gender and age. Model II expanded upon this by additionally controlling for socioeconomic and lifestyle factors such as educational attainment, income level, marital status, employment status, smoking, and alcohol consumption. Model III further refined the analysis by incorporating health-related variables into Model II, including body mass index (BMI), presence of activity limitations, perceived stress levels, subjective health status, and the presence of chronic diseases. 

According to the multiple logistic regression analysis results, the OR of depression in adults who slept <7 h was 1.65 (95% CI: 1.16–2.35) compared with that in those who slept 7–<9 h on weekdays. 

In Model I, the OR of depression in adults who slept >9 h on weekdays was 2.24 (95% CI: 1.18–4.28) compared with that in those who slept 7–<9 h on weekdays. However, the association was not significant in Models II and III. 

In terms of sleep duration on weekends, the adults who slept <7 h was 1.62 times (95% CI: 1.10–2.38) more likely to have depression than those who slept 7–<9 h. 

Furthermore, we divided the average sleep duration on into four groups, combining weekdays and weekends. The OR of depression in groups who slept <7 h on weekdays and <7 h on weekends was 1.76 (95% CI: 1.13–2.75) compared with those who slept 7–<9 h on weekdays and 7–<9 h on weekends.

## 4. Discussion

This cross-sectional study, utilizing nationally representative data, identified a significant association between insufficient average sleep duration (<7 h/day) on either weekdays or weekends and an increased likelihood of depression among Korean adults aged 19–64 years. These findings align with previous studies suggesting a link between short sleep duration and depressive symptoms.

A systematic review indicated that short sleep is associated with an increased risk of depression (RR = 1.13) [41], and a Korean study similarly reported higher rates of depressive symptoms among individuals sleeping ≤5 h compared to those with 7–8 h of sleep [42].

Sleep plays a vital role in neural plasticity and synaptic regulation [43]. Chronic sleep deprivation may impair these processes, thereby disrupting intra-and inter-regional communication within brain areas responsible for mood regulation [43,44]. Short sleep increases daytime sleepiness and fatigue, reducing physical performance and cognitive function [45] and negatively, it affects the prefrontal cortex, which is critical for executive function and emotional control [46]. According to an experimental study, acute sleep loss could lessen the tendency of obtaining positive information in a natural environment [44]. While several factors cause depression, repetition of negative thinking is associated with depression, which is affected by biases in emotional treatment and negative thinking when individuals acquire uncertain information [47]. Hence, persistent sleep deprivation is closely associated with negative thought patterns, which are in turn linked to the development of depressive symptoms. In other words, lack of sleep may act as a neurobiological stressor, excessively activating the stress response system and negatively affecting brain regions involved in the regulation of mood, emotion, and cognition, thereby contributing to the vulnerability to and maintenance of depression [22]. While inadequate sleep can be a key contributing factor to various health issues [48], the mechanism of its effects on depression remains unclear. The reason is that a sleep issue can be a sign of depression or an outcome of depression and the association between sleep issues and depressive symptoms is complicated [49]. As this study employed a cross-sectional design to examine associations, it is limited in its ability to establish causal relationships. Future research using longitudinal data will be necessary to clarify causal links.

Recently, sleep duration is affected by the social, economic, and technological changes. In particular, unstable labor, long-time work, increased environmental illumination, and increased use of mobile technology and social media have changed an individual’s lifestyle and affected his/her sleep [50]. Despite this, these factors could disturb one’s sleep duration and reduced sleep is generally a spontaneous and adjustable factor. According to a randomized experiment, sufficient sleep could reduce anxiety and depression by 20% [51]. Therefore, ensuring sufficient sleep should be considered a crucial strategy for preventing mental health problems, including depression.

According to the results of this study, even in Model III, which controlled for socio-demographic characteristics, individuals who slept less than seven hours on both weekdays and weekends showed the highest risk of depression. This group included a higher proportion of men than women, individuals in their 40 s and 50 s than other age groups, and married individuals compared to those who were unmarried. According to data from the Health Insurance Review and Assessment Service [52], middle-aged men account for 55.1% of male patients diagnosed with depressive disorders in South Korea [53], which may be related to South Korea’s status as one of the OECD countries with the longest working hours [54]. In particular, middle-aged men often engage in voluntary or unofficial overtime work to secure promotions or maintain their positions [55]. In the Korean cultural context, sleep deprivation among married middle-aged men is not merely the result of personal habits, but also stems from structural factors such as breadwinner responsibilities, extended working hours, and emotional restraint [56]. These findings highlight that intervention strategies should be tailored to the characteristics of reflect the sociocultural characteristics of the target population. To promote psychosocial well-being, individuals with short sleep should be identified as a vulnerable group and prioritized for customized interventions aimed at improving sleep-related risk factors. For middle-aged men in South Korea, sleep-based interventions should be tailored to reflect cultural norms such as long working hours, provider roles, and emotional restraint. Framing sleep hygiene and CBT in practical terms [57]—like improving focus or work performance—may increase acceptance. Digital self-monitoring tools (e.g., sleep apps or wearables) [58] offer discreet and autonomous support, aligning with preferences for private, self-directed health management among men. Above all, incorporating these strategies into workplace wellness programs can enhance accessibility and engagement, providing a culturally appropriate approach to preventing and managing depression in this high-risk group.

In contrast, our study did not identify a statistically significant association between long sleep duration (≥9 h) and depression. Previous studies have reported that both short and long sleep durations are associated with depressive symptoms [41], and a large-scale study conducted in the United States also demonstrated a U-shaped relationship between sleep duration and depression risk, with a significant increase in risk observed beyond 8 h of sleep [59]. The lack of association in our findings may be attributable to the relatively small number of participants reporting ≥9 h of sleep, or it may reflect cultural factors in South Korea, where extended sleep duration is generally not encouraged. Nevertheless, the potential health implications of long sleep, alongside short sleep, should not be overlooked. Future research incorporating objective measurements of sleep duration will be necessary to more accurately examine these associations.

A major strong point of this study is that it utilized a large, nationally representative sample to demonstrate the association between sleep duration and depression. Considering the complex sampling design that applied weights based on the KCDC guidelines, the results can be highly representative. While most studies used a single question about sleep in a 24-h cycle, our study used sleep duration that was divided into weekdays and weekends. Taking into account the substantial variation in sleep duration between weekdays and weekends is of great importance.

However, this study has some limitations. Many studies, including our study, investigate sleep duration using self-reported data; thus, the sleep duration could be underestimated compared to objective measurement, resulting in information bias. This study also failed to control vocational factors such as working hours and shift work, as well as hormonal imbalance factors such as menopause. Given that KNHANES does not assess the sleep quality—including sleep patterns, insomnia, and naps—in addition to sleep duration, certain aspects of the relationship between sleep and depression may not be adequately addressed in our study. Furthermore, generalizability to populations outside of Korea may be limited, given that the study sample was restricted to Korean individuals. This cross-sectional study cannot infer causal relations between sleep duration and depression, but it can show several meaningful associations that require further longitudinal research. In addition, more accurate results need to be obtained by using an objective method for the evaluation of sleeping habits.

Given that the factors contributing to depression may have changed before and after the COVID-19 pandemic [60], further research is warranted to re-examine the relationship between sleep and depression, considering these changes. Such studies would provide a more comprehensive understanding of how pandemic-related changes impact mental health outcomes and could inform the development of targeted interventions.

## 5. Conclusions

This study, which used nationally representative data from Korea, revealed that average sleep duration on weekdays and weekends is one of the factors related to depression. According to this study, an average sleep duration of less than 7 h on both weekdays and weekends is associated with depression. Therefore, we need to detect and cope with adjustable risk factors, with the objective of preventing and managing depression, especially in Koreans aged 19−64 years. As suggested by our findings, sleep duration should be considered as a factor for preventing depression. However, further longitudinal research using objective assessments of sleep habits is necessary, and other covariates not addressed in this study should be considered to obtain more accurate results.

## Figures and Tables

**Table 1:** Prevalence of depression according to participants’ general characteristics.

Variables	Total (*n* = 7669)	Depression		*χ^2^*	*p*-Value
		PHQ-9 ≥ 10 (*n* = 272)	PHQ-9 < 10 (*n* = 7397)		
Sex				13.718	<0.001
Male	3463 (53.1)	92 (2.6)	3371 (97.4)		
Female	4206 (46.9)	180 (4.4)	4026 (95.6)		
Age (years) *			41.2 ± 0.22	4.430	0.002
19–29	1344 (20.1)	67 (4.7)	1277 (95.3)		
30–39	1766 (19.6)	77 (4.2)	1689 (95.8)		
40–49	1932 (21.4)	55 (2.9)	1877 (97.1)		
50–59	1788 (19.1)	52 (2.5)	1736 (97.5)		
≥60	839 (8.0)	21 (2.2)	818 (97.8)		
Education				0.570	0.632
Elementary	497 (5.4)	23 (4.4)	474 (95.6)		
Middle school	578 (6.7)	25 (4.1)	553 (95.9)		
High school	2938 (40.2)	112 (3.4)	2826 (96.6)		
≥College	3656 (47.7)	112 (3.3)	3544 (96.7)		
Income level				12.210	<0.001
1st Quartile	622 (8.9)	49 (7.5)	573 (92.5)		
2nd Quartile	1819 (23.5)	80 (3.9)	1739 (96.1)		
3rd Quartile	2464 (32.5)	93 (3.6)	2371 (96.4)		
4th Quartile	2764 (35.2)	50 (2.1)	2714 (97.9)		
Marital status				16.013	<0.001
Married	5858 (70.3)	177 (2.8)	5681 (97.2)		
Single	1811 (29.7)	95 (5.0)	1716 (95.0)		
Smoking				9.318	<0.001
Current Smoker	1679 (24.8)	93 (5.1)	1586 (94.9)		
Ex-smoker	1263 (17.6)	40 (2.5)	1223 (97.5)		
Nonsmoker	4727 (57.7)	139(3.0)	4588 (97.0)		
Alcohol use				8.813	<0.001
High-risk alcohol use	1122 (15.6)	66 (5.7)	1056 (94.3)		
Proper alcohol use	3662 (49.5)	109 (2.9)	3553 (97.1)		
No alcohol use	2885 (34.9)	97 (3.3)	2788 (96.7)		
Physical activity				0.034	0.715
Yes	3626 (49.7)	125 (3.6)	3501 (96.4)		
No	4043 (50.3)	147 (3.4)	3896 (96.6)		
Economic activity				42.980	<0.001
Yes	5507 (72.3)	146 (2.4)	5361 (97.6)		
No	2162 (27.7)	126 (6.1)	2036 (93.9)		
BMI *				4.616	0.011
Underweight	315 (5.2)	18 (7.4)	297 (92.6)		
Normal weight	3878 (61.1)	137 (3.4)	3741 (96.6)		
Obesity	2111 (33.8)	70 (3.2)	2041 (96.8)		
Activity limitation				150.985	<0.001
Yes	45 (20.5)	201 (79.5)	246 (3.3)		
No	227 (2.9)	7196 (97.1)	7423 (96.7)		
Chronic disease				0.438	0.508
Yes	1361 (16.0)	51 (3.8)	1310 (96.2)		
No	6308 (84.0)	221 (3.4)	6087 (96.6)		
Stress				375.503	<0.001
Much	2143 (28.3)	232 (10.5)	1911 (89.5)		
Little	5526 (71.7)	40 (0.7)	5486 (99.3)		
Subjective health status				111.380	<0.001
Good	2569 (34.4)	24 (1.0)	2545 (99.0)		
Average	4108 (52.7)	120 (2.8)	3988 (97.2)		
Bad	992 (12.9)	128 (12.8)	864 (87.2)		
Average sleep duration on weekdays (hours) *			6.96 ± 0.17		
<7	3067 (40.5)	137 (4.5)	2930 (95.5)	9.435	<0.001
7–<9	4389 (56.4)	120 (2.6)	4269 (97.4)		
≥9	213 (3.1)	15 (6.3)	198 (93.7)		
Average sleep duration on weekends (hours) *			7.78 ± 0.20		
<7	1597 (20.3)	86 (5.3)	1511 (94.7)	4.274	0.015
7–<9	5194 (67.6)	146 (2.7)	5048 (97.3)		
≥9	878 (12.1)	40 (4.5)	838 (95.5)		

* Values are Mean ± SE (standard error); BMI: Body mass index; underweight: BMI < 18.5 kg/m^2^; normal weight: BMI 18.5–24.9 kg/m^2^; obesity: BMI ≥ 25 kg/m^2^.

**Table 2:** Participants’ general characteristics according to sleep duration on weekdays and weekends.

Variables	Total (*n* = 7669)	Average Sleep Duration on Weekdays (hours)			*χ^2^*	*p*-Value	Total (*n* = 7669)	Average Sleep Duration on Weekends (hours)			*χ^2^*	*p*-Value
		<7 (*n* = 3067)	7–<9 (*n* = 4389)	≥9 (*n* = 213)				<7 (*n* = 1597)	7–<9 (*n* = 5194)	≥9 (*n* = 878)		
Sex					1.425	0.244					6.836	0.001
Male	3463 (53.1)	1422 (41.5)	1952 (55.5)	89 (3.0)			3463 (53.1)	768 (21.7)	2325 (67.3)	370 (11.1)		
Female	4206 (46.9)	1645 (39.3)	2437 (57.5)	124 (3.2)			4206 (46.9)	829 (18.7)	2869 (68.0)	508 (13.3)		
Age (years)					9.551	<0.001					28.000	<0.001
19–29	1344 (20.1)	484 (35.7)	792 (59.1)	68 (5.2)			1344 (23.1)	172 (12.8)	918 (69.0)	254 (18.2)		
30–39	1766 (19.6)	608 (36.3)	1094 (59.7)	64 (4.0)			1766 (22.5)	255 (14.7)	1282 (72.1)	229 (13.3)		
40–49	1932 (21.4)	831 (44.6)	1071 (53.9)	30 (1.5)			1932 (24.5)	368 (19.7)	1359 (69.1)	205 (11.2)		
50–59	1788 (19.1)	803 (45.1)	953 (52.8)	32 (2.1)			1788 (21.9)	544 (30.7)	1109 (61.7)	135 (7.7)		
≥60	839 (8.0)	341 (40.8)	479 (57.3)	19 (1.9)			839 (8.0)	258 (30.5)	526 (63.2)	55 (6.3)		
Education					3.355	0.003					9.042	<0.001
Elementary	497 (5.4)	211 (44.0)	271 (52.8)	15 (3.1)			497 (5.4)	163 (31.2)	293 (60.6)	41 (8.2)		
Middle school	578 (6.7)	208 (34.8)	346 (60.4)	24 (4.8)			578 (6.7)	153 (26.8)	357 (61.1)	68 (12.1)		
High school	2938 (40.2)	1183 (40.1)	1658 (56.3)	97(3.7)			2938 (40.2)	629 (20.8)	1957 (66.4)	352 (12.7)		
≥College	3656 (47.7)	1465 (2.3)	2114 (100.0)	77 (56.4)			3656 (47.7)	652 (17.6)	2587 (70.3)	417 (12.1)		
Income level					5.467	<0.001					2.740	0.013
1st Quartile	622 (8.9)	230 (37.9)	352 (55.3)	40 (6.8)			622 (8.9)	134 (20.2)	393 (63.6)	95 (16.2)		
2nd Quartile	1819 (23.5)	710 (39.2)	1056 (57.5)	53 (3.4)			1819 (23.5)	405 (21.3)	1194 (65.5)	220 (13.2)		
3rd Quartile	2464 (32.5)	960 (39.6)	1434 (57.4)	70 (3.0)			2464 (32.5)	505 (20.8)	1689 (67.8)	270 (11.3)		
4th Quartile	2764 (35.2)	1167 (42.8)	1547 (55.2)	50 (2.0)			2764 (35.2)	553 (19.1)	1918 (69.9)	293 (11.1)		
Marital status					11.216	<0.001					34.849	<0.001
Married	5858 (70.3)	2382 (41.7)	3342 (55.9)	134 (2.4)			5858 (70.3)	1323 (22.5)	3959 (67.4)	576 (10.1)		
Single	1811 (29.7)	685 (37.6)	1047 (57.7)	79 (4.7)			1811 (29.7)	274 (15.0)	1235 (68.2)	302 (16.9)		
Smoking					1.469	0.210						
Current Smoker	1679 (24.8)	674 (41.8)	950 (54.4)	55 (3.8)			1679 (24.8)	391 (23.3)	1076 (63.2)	212 (13.5)	8.481	<0.001
Ex-smoker	1263 (17.6)	504 (39.9)	730 (57.5)	29 (2.5)			1263 (17.6)	306 (23.4)	846 (67.7)	111 (8.9)		
Nonsmoker	4727 (57.7)	1889 (40.1)	2709 (57.0)	129 (2.9)			4727 (57.7)	900 (18.0)	3272 (69.5)	555 (12.5)		
Alcohol use					1.521	0.195						
High-risk alcohol use	1122 (15.6)	445 (41.5)	647 (55.5)	30 (3.0)			1122 (15.6)	261 (23.7)	745 (65.2)	116 (11.0)	4.004	0.004
Proper alcohol use	3662 (49.5)	1414 (39.4)	2161 (57.8)	87 (2.7)			3662 (49.5)	691 (18.1)	2527 (69.5)	444 (12.5)		
No alcohol use	2885 (34.9)	1208 (41.5)	1581 (54.9)	96 (3.6)			2885 (34.9)	645 (21.8)	1922 (66.1)	318 (12.1)		
Physical activity					0.887	0.412					2.405	0.091
Yes	3626 (49.7)	1497 (40.8)	2045 (56.4)	84 (2.8)			3626 (49.7)	725 (19.1)	2485 (68.8)	416 (12.0)		
No	4043 (50.3)	1570 (40.1)	2344 (56.5)	129 (3.4)			4043 (50.3)	872 (21.4)	2709 (66.4)	462 (12.2)		
Economic activity					10.085	<0.001					1.608	0.201
Yes	5507 (72.3)	2239 (41.4)	3148 (56.1)	120 (2.5)			5507 (72.3)	1137 (20.2)	3713 (67.2)	657 (12.6)		
No	2162 (27.7)	828 (38.0)	1241 (57.3)	93 (4.7)			2162 (27.7)	460 (20.5)	1481 (68.6)	221 (10.9)		
BMI					6.434	<0.001					34.875	<0.001
Underweight	315 (5.2)	130 (41.0)	162 (51.0)	23 (8.0)			315 (5.2)	45 (13.9)	210 (66.5)	60 (19.6)		
Normal weight	3878 (61.1)	1485 (38.7)	2290 (58.2)	103 (3.1)			3878 (61.1)	738 (18.4)	2659 (68.4)	481 (13.2)		
Obesity	2111 (33.8)	895 (42.8)	1171 (54.8)	45 (2.4)			2111 (33.8)	510 (23.7)	1410 (66.9)	191 (9.4)		
Activity limitation					0.633	0.526					1.608	0.018
Yes	246 (3.3)	102 (39.1)	141 (59.2)	3 (1.7)			246 (3.3)	75 (28.7)	150 (62.0)	21 (9.3)		
No	7423 (96.7)	2965 (40.5)	4248 (56.3)	210 (3.1)			7423 (96.7)	1522 (20.0)	5044 (67.8)	857 (12.2)		
Chronic disease					10.605	<0.001					34.875	<0.001
Yes	1361 (16.0)	605 (45.3)	733 (53.3)	23 (1.4)			1361 (16.0)	390 (28.7)	877 (64.4)	94 (6.8)		
No	6308 (84.0)	2462 (39.6)	3656 (57.0)	190 (3.4)			6308 (84.0)	1207 (18.6)	4317 (68.2)	784 (13.1)		
Stress					19.936	0.001					15.560	0.004
Much	2143 (28.3)	944 (44.3)	1133 (52.4)	66 (3.2)			2143 (28.3)	499 (23.1)	1383 (65.0)	261 (11.9)		
Little	5526 (71.7)	2123 (39.0)	3256 (58.0)	147 (3.0)			5526 (71.7)	1098 (19.1)	3811 (68.7)	617 (12.2)		
Subjective health status					16.930	0.018					2.775	0.027
Good	2569 (34.4)	985 (38.7)	1521 (58.7)	63 (2.6)			2569 (34.4)	511 (19.1)	1755 (68.5)	303 (12.5)		
Average	4108 (52.7)	1649 (40.7)	2347 (56.1)	112 (3.2)			4108 (52.7)	836 (20.0)	2811 (68.1)	461 (12.0)		
Bad	992 (12.9)	433 (44.4)	521 (51.6)	38 (4.0)			992 (12.9)	250 (24.6)	628 (63.5)	114 (11.9)		

**Table 3:** Multiple logistic regression analysis of the association between depression and the sleep duration on weekdays and weekends.

Variables	Model I			Model II			Model III		
	OR *	95% CI	*p*-Value	OR *	95% CI	*p*-Value	OR *	95% CI	*p*-Value
Average sleep duration on weekdays (hours) (Ref: 7–<9)									
<7	1.88	(1.40–2.53)	<0.001	1.91	(1.41–2.58)	<0.001	1.65	(1.16–2.35)	0.005
≥9	2.24	(1.18–4.28)	0.014	1.65	(0.88–3.10)	0.120	1.68	(0.67–4.22)	0.267
Average sleep duration on weekends (hours) (Ref: 7–<9)									
<7	2.36	(1.69–3.29)	<0.001	2.08	(1.48–2.92)	<0.001	1.62	(1.10–2.38)	0.014
≥9	1.50	(0.99–2.29)	0.052	1.43	(0.93–2.20)	0.107	1.45	(0.80–2.61)	0.217
Average sleep duration on weekdays and weekends (hours) (Ref: 7–<9 and 7–<9)									
<7 and <7	2.66	(1.82–3.89)	<0.001	2.39	(1.61–3.53)	<0.001	1.76	(1.13–2.75)	0.013
≥9 and ≥9	2.45	(1.22–4.92)	0.012	1.74	(0.89–3.43)	0.108	1.74	(0.61–4.93)	0.300
Other groups	1.23	(0.89–1.71)	0.215	1.32	(0.94–1.85)	0.105	1.25	(0.83–1.88)	0.278

* Adjusted ORs from the multivariate logistic regression analysis. Model I: adjusted for age and sex; Model II: Model I + adjusted for education, income level, marital status, smoking, alcohol use, and economic activity; Model III: Model II+ adjusted for activity limitation, BMI, chronic disease, stress, and subjective health status. BMI: Body mass index; CI: confidence interval; OR: odds ratio.

## Data Availability

The original contributions presented in this study are included in the article. Further inquiries can be directed to the corresponding author(s).

## References

[1] World Health Organization Depression. https://www.who.int/news-room/fact-sheets/detail/depression.

[2] Goodwin R.D., Dierker L.C., Wu M., Galea S., Hoven C.W., Weinberger A.H. (2022). Trends in US Depression Prevalence From 2015 to 2020: The Widening Treatment Gap. Am J Prev Med..

[3] Health Insurance Review & Assessment Service Trends in the Medical Treatment of Depression and Anxiety Disorders (2017–2021). https://www.hira.or.kr/bbsDummy.do?pgmid=HIRAA020041000100&brdScnBltNo=4&brdBltNo=10627#none.

[4] Ko A., Kim K., Son J.S., Park H.Y., Park S.M. (2019). Association of Pre-Existing Depression With All-Cause, Cancer-Related, and Noncancer-Related Mortality Among 5-Year Cancer Survivors: A Population-Based Cohort Study. Sci Rep..

[5] Zis P., Daskalaki A., Bountouni I., Sykioti P., Varrassi G., Paladini A. (2017). Depression and Chronic Pain in the Elderly: Links and Management Challenges. Clin Interv Aging..

[6] Sarris J., O’Neil A., Coulson C.E., Schweitzer I., Berk M. (2014). Lifestyle Medicine for Depression. BMC Psychiatry..

[7] Hammen C. (2018). Risk Factors for Depression: An Autobiographical Review. Annu. Rev. Clin. Psychol..

[8] Jagannath A., Taylor L., Wakaf Z., Vasudevan S.R., Foster R.G. (2017). The Genetics of Circadian Rhythms, Sleep and Health. Hum. Mol. Genet..

[9] Hirshkowitz M., Whiton K., Albert S.M., Alessi C., Bruni O., DonCarlos L., Hazen N., Herman J., Katz E.S., Kheirandish-Gozal L. (2015). National Sleep Foundation’s Sleep Time Duration Recommendations: Methodology and Results Summary. Sleep Health.

[10] Wang X., Ma H., Gupta S., Heianza Y., Fonseca V., Qi L. (2022). The Joint Secular Trends of Sleep Quality and Diabetes Among US Adults, 2005–2018. J. Clin. Endocrinol. Metab..

[11] Kohyama J. (2021). Which Is More Important for Health: Sleep Quantity or Sleep Quality?. Children.

[12] Reiter J., Rosen D. (2014). The Diagnosis and Management of Common Sleep Disorders in Adolescents. Curr. Opin. Pediatr..

[13] Organization for Economic Co-operation and Development Balancing Paid Work, Unpaid Work and Leisure. https://web-archive.oecd.org/2015-10-08/268789-balancingpaidworkunpaidworkandleisure.htm.

[14] Lee S., Lee M., Seo B. (2022). The Effect of Sleep Duration on Obesity in Korean Adults. JCIT.

[15] National Health Insurance Service Tired Sleep Disorder Even After Sleeping, an Annual Average Increase of 8.1% for 5 Years. https://www.nhis.or.kr/bbs7/boards/B0039/31445.

[16] The Global Pursuit of Better Sleep Health. https://www.philips.com/c-dam/b2c/master/experience/smartsleep/world-sleep-day/2019/2019-philips-world-sleep-day-survey-results.pdf.

[17] McHill A.W., Wright Jr K.P. (2017). Role of Sleep and Circadian Disruption on Energy Expenditure and in Metabolic Predisposition to Human Obesity and Metabolic Disease. Obes. Rev..

[18] Besedovsky L., Lange T., Haack M. (2019). The Sleep-Immune Crosstalk in Health and Disease. Physiol. Rev..

[19] Wu Y., Zhai L., Zhang D. (2014). Sleep Duration and Obesity Among Adults: A Meta-Analysis of Prospective Studies. Sleep Med..

[20] Choi S.Y., Han J.E., Choi J., Park M., Sung S.H., Sung A.D. (2022). Association Between Sleep Duration and Symptoms of Depression Aged Between 18 and 49: The Korea National Health and Nutrition Examination Survey (KNHANES Ⅶ) From 2016 to 2018. Healthcare.

[21] Antza C., Kostopoulos G., Mostafa S., Nirantharakumar K., Tahrani A. (2022). The Links Between Sleep Duration, Obesity and Type 2 Diabetes Mellitus. J. Endocrinol..

[22] Li M., Wang N., Dupre M.E. (2022). Association Between the Self-Reported Duration and Quality of Sleep and Cognitive Function Among Middle-Aged and Older Adults in China. J. Affect Disord..

[23] Xu W., Tan C.-C., Zou J.-J., Cao X.-P., Tan L. (2020). Sleep Problems and Risk of All-Cause Cognitive Decline or Dementia: An Updated Systematic Review and Meta-Analysis. J. Neurol. Neurosurg. Psychiatry.

[24] Windred D.P., Burns A.C., Lane J.M., Saxena R., Rutter M.K., Cain S.W., Phillips A.J.K. (2024). Sleep Regularity Is a Stronger Predictor of Mortality Risk Than Sleep Duration: A Prospective Cohort Study. Sleep.

[25] Loprinzi P.D., Joyner C. (2018). Meeting Sleep Guidelines Is Associated With Better Health-Related Quality of Life and Reduced Premature All-Cause Mortality Risk. Am. J. Health Promot..

[26] Kim M.-Y., Lee S., Myong Y.H., Lee Y.J., Kim M.-R., Shin J.-S., Lee J., Ha I.-H. (2018). Association Between Sleep Duration and Stroke Prevalence in Korean Adults: A Cross-Sectional Study. BMJ Open..

[27] Sun X., Zheng B., Lv J., Guo Y., Bian Z., Yang L., Chen Y., Fu Z., Guo H., Liang P. (2018). Sleep Behavior and Depression: Findings From the China Kadoorie Biobank of 0.5 Million Chinese Adults. J. Affect. Disord..

[28] Vestergaard C.L., Simpson M.R., Sivertsen B., Kallestad H., Langsrud K., Scott J., Vedaa Ø. (2024). Weekday-to-Weekend Sleep Duration Patterns Among Young Adults and Outcomes Related to Health and Academic Performance. Sleep Sci. Pract..

[29] Kelly R.M., McDermott J.H., Coogan A.N. (2022). Differences in Sleep Offset Timing Between Weekdays and Weekends in 79,161 Adult Participants in the UK Biobank. Clocks Sleep..

[30] Cunha L.F., Santos R.B., Giatti S., Parise B.K., Aielo A.N., Silva W.A., Souza S.P., Bortolotto L.A., Lotufo P.A., Bensenor I.M. (2024). Gender Modulated the Association of Sleep Apnea and Sleep Duration With Arterial Stiffness: The ELSA-Brasil Study. Angiology.

[31] The Statistics on Korean National Health and Nutrition Examination in: Ministry of Health and Welfare. https://knhanes.cdc.go.kr/knhanes/main.do.

[32] Seeking Solutions: How COVID-19 Changed Sleep Around the World. https://www.philips.com/c-dam/b2c/master/experience/smartsleep/world-sleep-day/2021/philips-world-sleep-day-2021-report.pdf.

[33] Ciciurkaite G., Marquez-Velarde G., Brown R.L. (2022). Stressors Associated With the COVID-19 Pandemic, Disability, and Mental Health: Considerations From the Intermountain West. Stress Health.

[34] Negri A., Conte F., Caldiroli C.L., Neimeyer R.A., Castiglioni M. (2023). Psychological Factors Explaining the COVID-19 Pandemic Impact on Mental Health: The Role of Meaning, Beliefs, and Perceptions of Vulnerability and Mortality. Behav. Sci..

[35] Yang Q., Saleem M., Dobson E., Grimmesey S. (2024). The Mediating Role of Hesitancy in the Associations Between Mental Disorders and Social Support Seeking During the COVID-19 Pandemic. Behav. Sci..

[36] Deschênes S., Burns R., Graham E., Schmitz N. (2019). Depressive Symptoms and Sleep Problems As Risk Factors for Heart Disease: A Prospective Community Study. Epidemiol. Psychiatr. Sci..

[37] Moon H.M., Kim Y. (2020). Mental Health According to Sleep Duration in Stroke Survivors: A Population-Based Nationwide Cross-Sectional Study. Geriatr. Gerontol. Int..

[38] Kroenke K., Spitzer R.L., Williams J.B. (2001). The Phq-9: Validity of a Brief Depression Severity Measure. J. Gen. Intern. Med..

[39] Furihata R., Uchiyama M., Suzuki M., Konno C., Konno M., Takahashi S., Kaneita Y., Ohida T., Akahoshi T., Hashimoto S. (2015). Association of Short Sleep Duration and Short Time in Bed With Depression: Aj Apanese General Population Survey. Sleep Biol. Rhythms..

[40] Svensson T., Saito E., Svensson A.K., Melander O., Orho-Melander M., Mimura M., Rahman S., Sawada N., Koh W.P., Shu X.O. (2021). Association of Sleep Duration With All- and Major-Cause Mortality Among Adults in Japan, China, Singapore, and Korea. JAMA Netw. Open..

[41] Zhai L., Zhang H., Zhang D. (2015). Sleep Duration and Depression Among Adults: A Meta-Analysis of Prospective Studies. Depress Anxiety.

[42] Kim H.-M., Lee S.W. (2018). Beneficial Effects of Appropriate Sleep Duration on Depressive Symptoms and Perceived Stress Severity in a Healthy Population in Korea. Korean J. Fam. Med..

[43] Palagini L., Bianchini C. (2022). Pharmacotherapeutic Management of Insomnia and Effects on Sleep Processes, Neural Plasticity, a Brain Systems Modulating Stress: A Narrative Review. Front. Neurosci..

[44] Vargas I., Drake C.L., Lopez-Duran N.L. (2017). Insomnia Symptom Severity Modulates the Impact of Sleep Deprivation on Attentional Biases to Emotional Information. Cognitive Ther. Res..

[45] Ling A., Lim M.L., Gwee X., Ho R.C., Collinson S.L., Ng T.-P. (2016). Insomnia and Daytime Neuropsychological Test Performance in Older Adults. Sleep Med..

[46] Kayser K.C., Puig V.A., Estepp J.R. (2022). Predicting and Mitigating Fatigue Effects Due to Sleep Deprivation: A Review. Front. Neurosci..

[47] Hirsch C.R., Krahé C., Whyte J., Loizou S., Bridge L., Norton S., Mathews A. (2018). Interpretation Training To Target Repetitive Negative Thinking in Generalized Anxiety Disorder and Depression. J. Consult Clin. Psychol..

[48] Lauderdale D.S., Knutson K.L., Yan L.L., Liu K., Rathouz P.J. (2008). Sleep Duration: How Well Do Self-Reports Reflect Objective Measures? The Cardia Sleep Study. Epidemiology.

[49] Broström A., Wahlin Å., Alehagen U., Ulander M., Johansson P. (2018). Sex-Specific Associations Between Self-Reported Sleep Duration, Depression, Anxiety, Fatigue and Daytime Sleepiness in an Older Community-Dwelling Population. Scand J. Caring Sci..

[50] Covassin N., Singh P. (2016). Sleep Duration and Cardiovascular Disease Risk: Epidemiologic and Experimental Evidence. Sleep Med. Clin..

[51] Freeman D., Sheaves B., Goodwin G.M., Yu L.-M., Nickless A., Harrison P.J., Emsley R., Luik A.I., Foster R.G., Wadekar V. (2017). The Effects of Improving Sleep on Mental Health (Oasis): A Randomised Controlled Trial With Mediation Analysis. Lancet Psychiatry.

[52] Health Insurance Review and Assessment Service (2022, June 24). Analysis of Treatment Trends for Depression and Anxiety Disoders over the Past Five Years (2017–2021). https://www.hira.or.kr/bbsDummy.do?pgmid=HIRAA020041000100&brdScnBltNo=4&brdBltNo=10627.

[53] Kim K.H., Kim E.H. (2024). A Study on Middle-Aged Men’s Satisfaction With Directive and Non-Directive Counseling: Gender Role Conflict and Individualism-Collectivism. Korea J Counsel..

[54] Hours Worked: Average Annual Hours Actually Worked.

[55] Song Y.J., Lee Y.S. (2021). Work Hours, Work Schedules, and Subjective Well-Being in Korea. Int. Sociol..

[56] Kim J.Y., Yang Y., Bae S.H. (2022). Effects of Body Size Phenotype on Sleep Quality in Middle-Aged Korean Men. J. Men’s Health.

[57] Gkintoni E., Vassilopoulos S.P., Nikolaou G., Boutsinas B. (2025). Digital and AI-Enhanced Cognitive Behavioral Therapy for Insomnia: Neurocognitive Mechanisms and Clinical Outcomes. J. Clin. Med..

[58] Guillodo E., Lemey C., Simonnet M., Walter M., Baca-García E., Masetti V., Moga S., Larsen M., Ropars J., Berrouiguet S. (2020). Clinical Applications of Mobile Health Wearable–Based Sleep Monitoring: Systematic Review. JMIR Mhealth Uhealth.

[59] Dong L., Xie Y., Zou X. (2022). Association Between Sleep Duration and Depression in US Adults: A Cross-Sectional Study. J. Affect Disord..

[60] Ceolin C., Limongi F., Siviero P., Trevisan C., Noale M., Catalani F., Conti S., Di Rosa E., Perdixi E., Remelli F. (2024). Changes in Sleep Duration and Sleep Timing in the General Population From Before to During the First COVID-19 Lockdown: A Systematic Review and Meta-Analysis. Int. J. Environ. Res. Public Health.

